# Role of the T and B lymphocytes in pathogenesis of autoimmune thyroid diseases

**DOI:** 10.1186/s13044-018-0046-9

**Published:** 2018-02-13

**Authors:** Marta Rydzewska, Michał Jaromin, Izabela Elżbieta Pasierowska, Karlina Stożek, Artur Bossowski

**Affiliations:** Department of Pediatrics, Endocrinology and Diabetes with a Cardiology Unit, Medical University of Bialystok, ul. Waszyngtona 17, 15-274 Białystok, Poland

**Keywords:** Autoimmune thyroid diseases, T helper, T regulatory, Graves’ disease, Hashimoto’s thyroiditis

## Abstract

Autoimmune thyroid disorders (AITD) broadly include Graves’ disease and Hashimoto’s thyroiditis which are the most common causes of thyroid gland dysfunctions. These disorders develop due to complex interactions between environmental and genetic factors and are characterized by reactivity to self-thyroid antigens due to autoreactive lymphocytes escaping tolerance. Both cell-mediated and humoral responses lead to tissue injury in autoimmune thyroid disease. The differentiation of CD4+ cells in the specific setting of immune mediators (for example cytokines, chemokines) results in differentiation of various T cell subsets. T cell identification has shown a mixed pattern of cytokine production indicating that both subtypes of T helper, Th1 and Th2, responses are involved in all types of AITD. Furthermore, recent studies described T cell subtypes Th17 and Treg which also play an essential role in pathogenesis of AITD. This review will focus on the role of the T regulatory (Treg) and T helper (Th) (especially Th17) lymphocytes, and also of B lymphocytes in AITD pathogenesis. However, we have much more to learn about cellular mechanisms and interactions in AITD before we can develop complete understanding of AITD pathophysiology.

## Introduction

Graves’ disease (GD) and Hashimoto thyroiditis (HT) are classified as autoimmune thyroid disorders (AITD) characterized by the breakdown of self-tolerance to thyroid antigens resulting in circulation of antibodies and lymphocyte infiltration [1]. There is a growing trend for the prevalence of Hashimoto’s disease and it is estimated at approximately 5-10% [2, 3]. Women are diagnosed with it five to ten times more often than men and its frequency increases with the age (the highest number of cases is observed between 45 and 65) [3]. In pediatric population, the most common age at presentation is adolescence’ yet, HT may develop at any time, rarely even in infants [4, 5]. In case of GD, meta-analyses of different studies have estimated the general frequency of the disease to be about 1% [6]. However, Graves’ disease is four to five times more common in women than in men [7].

The pathogenesis of HT is basically the result of cell-mediated autoimmune, whereas GD results from humoral autoimmunity [8, 9]. However, as in other autoimmune disorders, in AITD, humoral and cellular immune mechanisms are closely connected and cross-linked [10]. Moreover, in both diseases, the thyroid cell itself takes part in the intrathyroidal immune process. Classically, HT is considered to be a Th1-mediated disease’ yet, this classification has altered due to the description of new Th cell subsets including Th17 cells [11]. In accordance with the newest research findings, uncontrolled Th17 cell responses have been involved in different types of autoimmune diseases, which were previously supposed to be Th1-dependent diseases [12]. In HT, as a consequence, chronic inflammatory cell infiltrates into the thyroid gland, which includes predominantly thyroid-specific B and T cells. In result, goiter may initially be caused [13]. Subsequently, hypothyroidism, the characteristic hallmark of thyroiditis, can develop when sufficient numbers of follicular cells responsible for the production and secretion of thyroid hormones thyroxine (T4) and triiodothyronine (T3) are destroyed [14]. It is now well established that GD, B, and T lymphocyte-mediated autoimmunity are known to be directed against four well-known thyroid antigens: thyroglobulin (Tg), thyroid peroxidase (TPO), sodium-iodide symporter (NIS) and the thyrotropin receptor (TSH-R) [15, 16]. However, the THS-R itself is the main autoantigen of GD [17]. The circulating agonist autoantibodies against the TSH-R (TRAb), which bind to and activate the receptor, they, thereby, chronically stimulate thyroid hormone synthesis and secretion (causing hyperthyroidism) as well as thyroid hyperplasia and (causing a diffuse goiter) [18]. Furthermore, TRAb, particularly in children, strongly stimulate thyroid function and have prognostic significance, probably as costimulatory molecules [19, 20]. Thyroid-associated ophthalmopathy occurs in around 25% of cases and is the most common extrathyroidal manifestation of GD [21, 22]. Furthermore, both the humoral and cellular immune actions seem to be present in its pathogenesis [22].

The majority of researchers share the opinion that AITD are multifactorial diseases, caused by a complex interplay of genetic, hormonal, and environmental influences that provoke the development of inappropriate immune responses against thyroid on multiple levels and the initiation of a long-standing autoimmune reaction [23–25]. This review will focus on the role of the T regulatory (Treg) and T helper (Th) (especially Th17) lymphocytes, and also of B lymphocytes in AITD pathogenesis. However, we have much more to learn about cellular mechanisms and interactions in AITD before we can develop complete understanding of AITD pathophysiology.

### T lymphocytes

T lymphocytes originate from precursor stem cells in fetal liver and bone marrow and differentiate into mature cell types after migration to the thymus [26]. T lymphocytes may be categorized based on their distinct function into cytotoxic T lymphocytes (expressing the surface protein cluster of differentation (CD) 8 and responsible mainly for immune defence against intracellular pathogens and for tumour surveillance) and helper T lymphocytes (expressing the surface protein CD4) [27]. In this review, we focus on CD 4+ cells. Helper T cells (naïve CD4+ T lymphocytes) are triggered when they are presented with peptide antigens by MHC (major histocompatibility complex) class II molecules, which are expressed on the professional antigen-presenting cells (APCs) surface. Both are necessary for production of an adequate immune response [28]. T cells have on their surface T cell antigen receptors (TCR) responsible for recognition of an antigen/major histocompatibility complex (HLA complex), immunological accessory molecules identifying HLA determinants, and adhesion molecules recognizing their counterpart ligands on APCs [29, 30].

**Fig. 1:**
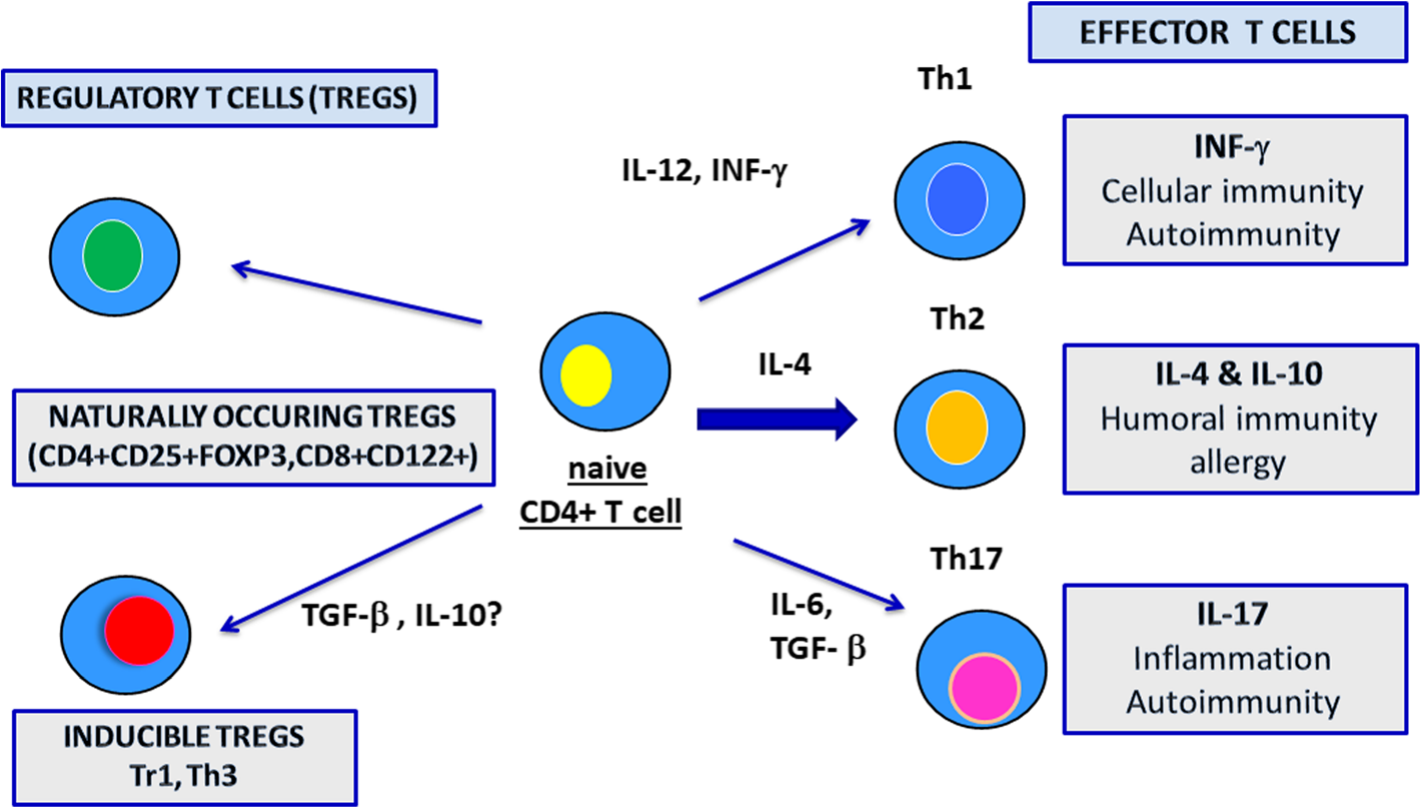
The differentiation of CD4+ cells into specific T cell subsets. Cytokines play crucial roles in determining Th cell differentiation and the combination of cytokines is required for the differentiation of each subset

Once activated, helper (CD4+) T cells can be subdivided into at least three main functional subtypes according to releasing cytokines, the Th1 subset (mainly involved in cell–mediated tissue-damaging reaction), the Th2 subset (driving B cells to produce antibodies in the humoral immune response), and Th17 cells (playing a role in immune responses to infectious agents and maintenance of autoimmune diseases) [31, 32]. Th 1 cells produce tumor necrosis factor-β (TNF- β), interferon gamma (IFN-γ), and interleukin (IL) 2,;Th 2 cells secrete mainly IL-4, IL-5, IL-6, and IL-13, and Th17 secrete IL-17 [27, 28, 33] (Fig. 1). Moreover, some CD4+ T cells produce both Th1 and Th2 cytokines and have been termed Th0 [34]. Determination of Th subtype is activated during an immune response, which depends on the type of antigen and its concentration, the nature of the initial antigen-presenting cell, and, probably, on ill-defined genetic and environmental influences [35]. We recognize also set of T cells that can suppress these inflammatory responses, described as regulatory T cells (T regs) [32, 36] (Fig. 1).

The particular factors beginning thyroid autoimmunity are not well known, but many potential influences have been described. Development of autoimmunity is, probably, as mentioned, a process including both genetic and environmental effects [23–25]. Immunologic self-tolerance is induced during the perinatal period, when immature lymphocytes in the thymus are exposed to self-antigens [37, 38]. At this crucial moment, clonal deletion or induced anergy of autoreactive T cells determines self-tolerance to autoantigens. However, these mechanisms are never ideal and some autoreactive cells may be normally present in their circulation [39]. As we described above, AITD arise from a breakdown of self-tolerance to thyroid antigens and this process might be induced by excessive exposure to thyroid antigens, a modified self-antigen, exposure to environmental antigens that mimic a self-antigen, polyclonal immune activation, or idiotypecross-reaction of self-antigens [40].

### Th1 and Th2 lymphocytes

The relationship between GD and HT has been discussed for many years. The familial association with GD and the fact that it may sometimes develop into HT (and vice versa) seem to signify that the two disorders are closely related pathophysiologically, although not functionally [41, 42]. Mixed mRNA (messenger ribonucleic acid) pattern of cytokine secretion revealed that both the Th1 and Th2 subtypes of the helper T cell responses are involved in both Graves’ disease and Hashimoto’s thyroiditis [43]. In HT Th1, lymphocytes activate a strong lymphocyte inflammatory infiltrate of the thyroid, which causes subsequent thyroiditis and thyroid gland damage. This is caused by activation of cytotoxic lymphocytes and macrophages, which directly affect thyroid tissue by destroying thyroid follicular cells [44]. Furthermore, in HT, thyroid follicular cells are induced to express functional Fas receptor and also Fas ligand (two molecules involved in the regulation of programmed cell death) by cytokine stimulation from APCs and Th1 cells (mainly interleukin-1). This mechanism may cause self-apoptosis [45]. On the other hand, Th2 induce an excessive stimulation and production of B cells and plasmatic cells, which produce antibodies against thyroid antigens leading also to thyroiditis [46]. In fact, although HT is characterized by cell-driven destruction to thyroid cells, autoantibodies to TPO and Tg are also a crucial component in its pathogenesis [47].

The misfunction of Th1subtype of lymphocytes has also been reported in GD, which contradicts, as we mentioned, the traditional view that HT was caused by Th1-mediated cellular immune mechanisms and GD by humoral Th2-mediated mechanisms only [42]. Most Graves’ patients also have detected in blood tests autoantibodies to TPO and, less frequently, to Tg [48]. Th1 cytokines induce the production of subclass immunoglobulin (Ig) G1, whereas Th2 cytokines drive the generation of subclass IgG4 [49]. Furthermore, the early phase of a humoral immune response generally includes IgG1, whereas presence of antibodies to subclass IgG4 is associated with long-standing immunization, TPO and Tg autoantibodies may comprise IgG4 as well as IgG1 subclasses, indicating participation of Th2 and Th1 cytokines [50]. Moreover, it was assessed that the stimulating activity of thyrotropin receptor antibodies found mostly in the IgG1 subclass, which is selectively induced by Th 1 cells [51]. It is also worthwhile noting that Th1 cells may also induce antibody production through secretion of IL-10, which, in turn, activates B cells [52]. In case of GD, persistent increased levels of autoantibodies directed against TSHR stimulate the growth and function of thyroid follicular cells, thus, leading to development of goiter and hyperthyroidism [18]. To sum up, IgG1 antibodies (such as Th1-related TSAb) arise early in the immune response, whereas IgG4 antibodies (to TPO and Tg, typically Th2-related) arise after prolonged immune stimulation. These conclusions are consistent with the clinical manifestations of GD and HT [53]. Furthermore, one of the newest research papers described the level of mRNA expression for the genes encoding T-bet and GATA3 (main regulators of the Th1 and Th2 differentiation, respectively) together with Th1 (IFN-γ) and Th2 (IL-4) cytokine mRNA expression in patients with GD. The researchers indicated that in comparison with the control group, T-bet and IFN-γ mRNA expression levels were significantly up-regulated in the GD patients, while GATA3 and IL-4 mRNA expression levels were downregulated. Their results showed that a Th1/Th2 imbalance is present in GD, and it may be involved in the pathogenesis of disease [54].

### Th17 lymphocytes

It has been noted, that preferential generation of various Th cell subpopulations also includes conversion to subset called Th17 lymphocytes [55], which were discovered and described for the first time in 2003 [56]. This linage of lymphocytes is characterized by production of cytokines from IL-17 family [57], which ultimately contributes to exacerbation of autoimmunologic process [58, 59]. Moreover, their role in pathogenesis of AITD was confirmed by latter research [60].

Although the dissonant data in a matter of Th17 lymphocytes’ differentiation has been published so far, it is suspected to be dependent on simultaneous co-occurrence of few factors. Such factors are activation of intracellular pathway called Signal Transducer and Activators of Transcription-3 (STAT3), presence of cytokines (such as IL-6, IL-Beta and IL-23), and high expression of transcription factor receptor-related orphan receptor C2 (RORC2) on the cells’ surface [61–63]. So called classical or conventional Th17 lymphocytes are specified with production and excretion of IL-17A, IL-17-F, IL-21 and IL-22 [64], which main role is intensifying release of other proinflammatory cytokines like IL-Beta, TNF-Alfa, and chemokines. Their crucial role is stimulation of vessel-related cells (epithelial cells) or connective tissue cells (fibroblasts and macrophages) that are primarily involved in causing tissue damages seen in autoimmune inflammatory conditions. Accordingly, further analyses considering Th17-associated levels of cytokines have been carried out. Thus, it was reported that levels of mentioned cytokines such a IL-21, IL-22 and IL-23 have been considerably raised in patients with AITD, especially in HT [63, 65–69]. Moreover, conventional Th17 under sustained exposure to different cytokines may subsequently differentiate to cells performing varied functions, proclaiming high plasticity of this subpopulation. Thus, under prolonged influence of IL23, they turn into specialized pathogenic cells called Th1-like (non-classic Th1), able to synthesize IFN-gamma and granulocyte-macrophage colony-stimulating factor (GMCSF), whereas losing the ability of producing IL-17 [70]. Noted cell type undoubtedly has a very relevant impact on the pathogenesis of molecular damages in course of AITD [71–74].

Concerning pathogenesis of AITD, molecules suspected to regulate functions of Th17 have been detected as follows: leptin (inductor, affecting directly naive T cells), GITRL (inductor, inhibiting functions of Tregs) [59, 75], and galectins, especially type 9 (inhibitor) [76]. In peripheral blood and thyroid inflammatory environment in patients with AITD, especially HT, higher concentration of Th17 and their excreted cytokines were observed. These patients were characterized by higher transcriptional activity of RORC2 gene, affecting with more intense conversion into Th17 cells in-vitro [59, 77]. Additionally, higher levels of Th17+ T cells and lower proportion Tregs to Th17 were observed in these patients suffering from Hashimoto [75, 76]. Considerable impact on progress of AITD have an imbalance between Th17 lymphocytes and Tregs, which seems to be very important in development of the disease [59, 78, 79]. T regulatory cells are responsible for control of autoimmune process, while Th17 cells support autoimmune activities. The Th17/Treg ratio was remarked significantly higher in patients with HT compared with healthy controls and the correlation between the levels of GITRL and the proportion of Th17 cells was found positive [59]. The described positive correlation may be caused by GITRL inhibiting function towards Tregs (see above). Moreover, in patients suffering from HT, there have been also discovered unusually high levels of Th17 cells and Th-17-associated proinflammatory cytokines [79] both in thyroid tissue and/or in peripheral blood which has been confirmed in subsequent assays [80–82]. Additionally, intensified in-vitro conversion of their T cells to Th17 was observed [66]. Altogether, it is clear that Th17 lymphocytes play a key role in development and progress of AITD [66, 83], which makes them plausible potential aims for innovative immunosuppressive treatment. The antagonists of Th17 cells such as anti-IL-23, anti-IL-17, anti-IL-6R mAbs, chemokine blockers, or STAT3 inhibitors create a promising possibility of application to patients suffering from AITD.

### T regulatory (Treg) cells

Among many diversified types of cells taking part in pathophysiological process of AITD, the unique subtype of T lymphocytes, namely T regulatory cells (Tregs), was observed [32, 36]. Although their potential role in immunosuppressive processes has been already noted primarily by Gershon and Kondo in 1970 [84], which has been confirmed by subsequent scientific assays [85–87] the interest in the field of suppressor T cells gradually raises and they remain the main subject of various scientific investigations. T regulatory cells, belonging to T helper CD3+ CD4+ cells family, are divided into 5 extensive groups basing on expression of different molecules on their surface; thereby, they promote in each specific immunosuppressive such features as CD4 + CD25+ FoxP3+ natural (or constitutive) T regulatory cells (nTregs), CD4+ CD25+ FoxP3+ inducible (or adaptive) T regulatory cells (iTregs), peripheral (pTregs), Tr1 type Tregs (IL-10 dependent), Th3 type Tregs (TGF-alfa dependent, LAP+), and CD8+ Tregs [88]. First two subpopulations of Tregs are best known due to numerous scientific assays. In contrast, three recent groups were narrowly investigated and remain another relevant issue to be addressed in patients with AITD [89]. Furthermore, according to report of Cortes et al., new FoxP3+ CD69+ Treg subset, responsible for maintaining tolerance and protection of developing inflammation, was recently discovered. Interestingly, in regard to another assay, their immunosuppressive functions were impaired in large part of patients with AITD. Natural T regulatory cells are believed to be crucial element in maintaining peripheral tolerance in vivo through the active suppression of self-reactive T-cell activation and expansion [89–91]. They are identified by high levels of CD25 whose specific constitutional expression is unique for vast majority of these cells and the IL-2 receptor (IL-2R) [92, 93]. These two molecules are making Tregs easy to be identified by multi-parametric flow cytometry. Secondly, specified subpopulation of Foxp3+ Tregs, called pTregs, can also be developed from naïve T cells. Indispensable condition for this process is stimulation in presence of cytokines such as TGF-b and IL-2 [94, 95].

**Fig. 2:**
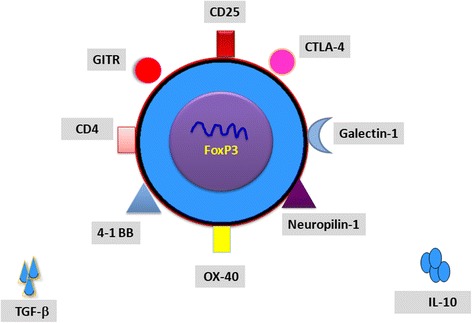
Schematic representation of Treg cell structure. CD25, cluster of differentiation 25; CD4, cluster of differentiation 4; CTLA-4, cytotoxic T cell antigen 4; OX-40, tumor necrosis factor receptor superfamily, member 4; 4-1 BB, tumor necrosis factor receptor superfamily member 9; GITR, tumor necrosis factor receptor superfamily member 18

Furthermore, basing on past scientific reports, it has been noted that all Treg cells, mostly with expression of FoxP3, contribute to prevention and pathogenesis of autoimmune diseases, including AITD [63, 96, 97]. Mentioned contribution of Tregs was subsequently confirmed by development of AITD in humans that have qualitative or quantitative disturbances of Foxp3 expression [98–101] and lesser concentration or lack of pTregs and tTregs [102, 103]. Considering both tTregs and pTregs, it was observed that they have a tendency to accumulate in inflammated thyroid tissue [97, 103–105], whereas their count in peripheral blood widely varies in patients suffering from AITD. Nevertheless, it is clear that they are dysfunctional and unable perform their immunosuppressive functions in afflicted subjects [99, 102, 106]. Presumably, the reason for that phenomenon might be connected with possible conversion of Tregs to pro-inflammatory cells (mainly Th17and Th1 lymphocytes) [107–109], in the presence of different cytokines in thyroid’s inflammatory microenvironment (IL-2/IL-15 and IL-21/IL-23) [110, 111]. Regardless, there is a need of conducting further studies to prove this thesis. Development of AITD also depends on vulnerability of immune-regulatory genes and their final products of transcription, which are located on the surface of T regulatory cells such as FOXP3, CD25, and CLTA-4 (Fig. 2). These genes and their products are involved in ensuring proper maintenance of peripheral tolerance (FOXP3 and CD25) and establishing an appropriate co-stimulation (CLTA-4). Due to various factors, improper activity of these immune-regulatory genes would potentially lead to a breakdown in immune tolerance and ultimately autoimmunologic disease such as AITD [112]. Forkhead box P3 (FOXP3) is a gene located on chromosome X (Xp11) [113]. T regulatory cells are characterized by an expression of FOXP3, whose involvement in the processes of differentiation, maintenance, and function of Treg cells is essential [99, 114–117]. In humans, disruptions in FOXP3 gene or deficiencies of this molecule lead to the syndrome comprising immune dysregulation, polyendocrinopathy and enteropathy (IPEX) [118–120], which is more common in men. Diverse polymorphisms of FOXP3 gene have been reported to be corresponding with autoimmune thyroiditis (AITD) [121, 122]. CD25 (the α-chain of the IL-2R, IL-2Rα receptor,) primarily intercedes IL-2 signaling, which is essential for providing survival and growth to CD25 + CD4+ Tregs [123]. Thereby, it is potentially possible that some genetic changes in the CD25 gene predispose to development of an immunological process by disarranging Treg function and adequate development of peripheral tolerance.

CTLA-4 (also called CD152) is a transmembrane protein. Upon T cell activation, ligation of CTLA-4 on the surface of Tregs with its ligands (CD80 and CD86) APCs, competing CD28 (protein responsible for co-stimulatory signals enabling full activation of T cells), contributes to diminishing of T-cell activation, reduced IL-2 production and the arrest of T cell cycling, and further activation. Basing on subsequent findings, it has been presumed that CTLA-4 may perform the key role in regulation of central Treg-mediated suppressive mechanism. The presence of CTLA-4 is originally restricted to the surface of CD4 + CD25+ T cells [124–126] in normal mouse models was constructively expressed by CD25+ CD4+ T cells [127–130], also in humans [131]. Additionally, several variants of single nucleotide in CTLA-4 gene have been connected with risk for thyroid autoimmunity, including both Graves’ Disease and Hashimoto thyroiditis [112, 132–135]. Secondly, blockade of CTLA-4 with monoclonal antibodies, was not expressed by responder T cells, but only by natural Tregs, repealing their immunosuppressive abilities [124]. Intimately, analysis of transcription factors’ activity such as FOXP3 revealed that they up-regulate CTLA-4 expression by binding to it’s gene promoter region [124], which indicates codependency and cooperation of these mechanisms and their importance in Tregs functioning.

More often, Tregs are considered as a plausible candidate for therapeutic aim in patients with AITD. It has been reported in various studies that presented by iTregs (in-vitro indicated Tregs) state of instability (i.e no signs of proliferation, production of effector cytokines such as IL-2, TNF-α, IFNγ, or IL-4 upon stimulation via their TCR), presumably, as a result of worsened stabilization of Foxp3 expression, differs from nTregs. There is a possibility that the differences in epigenetic modifications contribute to this phenomenon in iTregs. This important remarking for the present complicates or unables the utilization of ex vivo-expanded iTregs for adoptive immunotherapy [95, 136]. Therefore, a mechanism, which enables conversion of unstable into stable iTregs in vivo environment, must be discovered. This requirement is crucial for generating cells suitable for treatment application. Notably, a thorough examination of modifications on epigenetic level in the future assays may help in generating iTregs useful for clinical purposes [95].

### B lymphocytes

B lymphocytes develop from hematopoietic stem cells. Maturation of B cells takes place in bone marrow, whereas their activation occurs in the secondary lymphoid organs such as lymph nodes and the spleen [137].

B cells represent mainly the humoral immunity. Nevertheless, their role as a cell itself is equally relevant. In practise, they are activated in patients with AITD [3]. In Graves’ Disease, B cells play a vital role as they are the source of pathognomonic activating autoantibodies (TRAb) against thyroid-stimulating hormone receptor (TSHR) [10]. TRAb, by binding to the receptor, chronically stimulates it. TSHR is expressed on thyroid follicular cells; thus, the consequence of this chronic stimulation is an increased production and secretion of thyroid hormones T_4_ and T_3_ [43, 138]. Although the role of B cells in development of Hashimoto’s thyroiditis is not as significant as in GD, it should be mentioned that they produce autoantibodies to the thyroglobulin (Tg) and thyroid peroxidase (TPO), which are thyroid self-antigens [10]. Antibody-dependent cell-mediated cytotoxicity is a meaningful factor responsible for apoptosis of thyroid follicular cells in HT.

B cells can also serve as APCs. They have a transmembrane receptor, called BCR (a surface immunoglobulin), which enables them to identify specific antigens, against which they initiate an immune response and synthesize antibodies, and present fragments of these antigens to CD4+ T cells using MHC class II molecules [10, 139]. When the antigen is uncommon, B cells may be the dominant APCs as they have an ability of up-concentration antigens on the cell due to the presence of BCR in the cell membrane [140]. T helper (Th) cells reciprocally support activation of B cells. Particular attention was paid to sequencing of thyroid antibodies and defining B cell epitopes in TSH receptor. This, in turn, could enable further understanding of the pathogenesis of GD, which is a cause of triggering TSHR leading to development of this disease [141]. However, the pace of the autoimmune reaction in AITD is usually slow, which leads its proliferation and differentiation involving many different polyclonal B and T cells [10].

B cells exert their activity of antibody synthesis in the thyroid gland. Moreover, intense autoreactive B cell infiltration of the thyroid tissue is observed in patients with AITD [10, 142]. It means that the thyroid may be a major place for thyroid antibody secretion and presents a significant role in promoting persistence of AITD. Additionally, reduced serum concentration of thyroid antibody after surgical or radioiodine thyroid ablation or any other antithyroid drug treatment, is a confirmation of this finding [10, 143]. There are studies showing that anti-CD20 therapy efficiently depletes B cells in thyroid glands of mice with autoimmune thyroiditis, even though many of thyroid B cells do not express CD20 [144]. Moreover, the fact that the therapy using rituximab, B cell-depleting anti-CD20 antibody, induces clinical improvement in Graves’ ophthalmopathy, suggests a crucial role of B cell involvement and provides a base for the development of new therapeutic strategies in patients suffering from AITD [145].

B cells not only participate in proinflamatory reactions. They also play a role in regulation of immune responses. Recent studies identified regulatory B (Breg) cells as specific subsets that have an ability of immune response suppression [143]. They contribute to maintenance of peripheral tolerance and inhibition of immune reaction to specific self-antigens, mainly by producing of interleukin-10 (IL-10) but also by transforming growth factor (TGF-β), Fas ligand, and expressing of TNF-related apoptosis-inducing ligand (TRAIL) [138, 146].

There are multiple subsets of IL-10-producing Breg cells that have been described in the studies. In mice, these include marginal-zone (MZ) B cells, transitional 2 marginal-zone precursor (T2-MZP) cells, Tim-1+ B cells, CD5^+^CD1d^hi^ B (B10) cells, CD138^+^ plasma cells, and plasmablasts [143]. In humans, CD19^+^CD24^hi^CD27^+^ and CD19^+^CD24^hi^CD38^hi^CD1d^hi^Breg cells have been described [143]. In latest studies, it has been demonstrated that both in mice and humans, immature B cells, mature B cells, and plasmoblasts have an ability to differentiate into Breg cells [143]. These findings support the conception that the primary condition for Breg cell differentiation is not the expression of a factor specific to Breg cell lineage, but rather the environment in which a B cell is located.

**Fig. 3:**
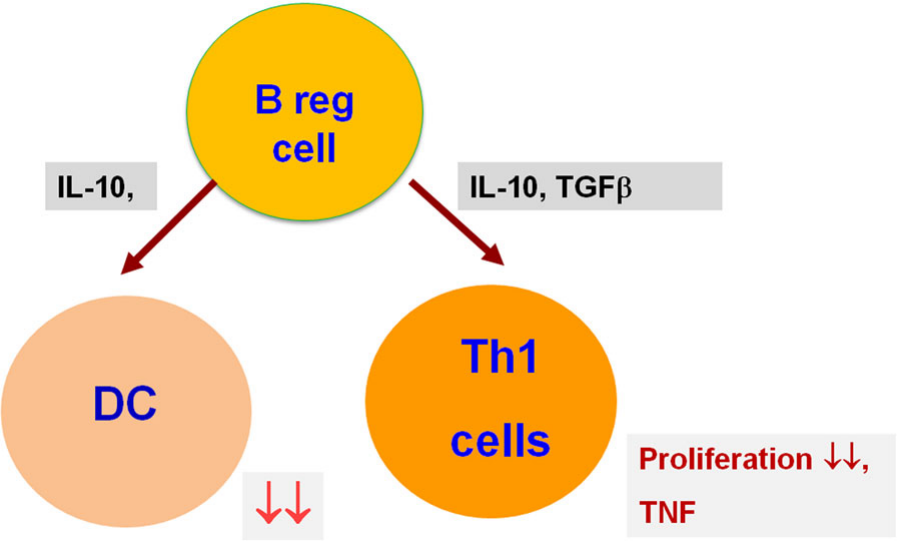
Schematic representation of Breg cells function. Through the production of IL-10 and TGF-β Breg cells can suppress the differentiation of pro-inflammatory lymphocytes and maintain of self-tolerance. DC, dendritic cells, IL-10, interleukin-10; TGFb, transforming growth factor β; TNF, tumor necrosis factor

On the ground of a number of data, Breg cells are important in preventing the disease onset and also in suppressing the disease symptoms. Primarily, Breg cells are able to change T cell differentiation in behalf of a regulatory phenotype [143] (Fig. 3). It is considered that related interactions between Breg cells and T cells control the induction of T regulatory (Treg) cells and are important in maintaining Treg cell compartment. There are studies showing that population of Treg cells is reduced in mice with B cell deficiency [143]. According to recent findings, Breg cells have an ability to inhibit Th1 immune responses by the production of IL-10 during chronic infections [143]. Furthermore, they are capable of indirect suppression of Th1 and Th17 cells differentiation by suppressing production of pro-inflammatory cytokines by dendritic cells [140, 143]. Production of TGF-β by Breg cells enables them to induce both anergy in CD8+ and apoptosis of CD4+ effector T cells [143]. Microbiota also plays a role in Breg cells generation. In murine studies, population of Breg cells was decreased in mice treated by antibiotics in comparison to untreated individuals [147]. There are also data showing Breg cells dysfunction in human diseases. According to them, chronic exposure of B cells to elevated inflammatory cytokines levels leads to reduction of the functional Breg cells population, which are unable to restore the peripheral tolerance [146, 148]. Moreover, researchers from our university conducted the study which showed a significant decrease of CD19^+^CD24^hi^CD27^+^IL-10^+^ and CD19 + IL-10^+^ B lymphocytes in untreated patients with GD and HT in comparison to the healthy controls. They concluded that the reduction number of Breg cells with expression of CD19^+^CD24^hi^CD27^+^IL-10^+^and CD19^+^IL-10^+^(B10) could be responsible for loses immune tolerance and development of autoimmune process in thyroid disorders [149].

Furthermore, the issue of therapeutic potency of Breg cells is also raised in the studies. According to them, future treatment of AITD should be focused on restoration of immune tolerance. Kristensen in her study [140] suggests some future experiments that could enable better understanding of the pathogenesis of AITD, including measuring the functionality of the IL-10 produced by Breg cells or characterization of the phenotype of these cells in the thyroid tissue in patients with AITD. Clarification of more details of the immune responses regulation by Breg cells could provide a ground for the development of new B cell-mediated therapeutic strategies in patients with AITD [138, 146, 147].
